# Robust, real-time generic detector based on a multi-feature probabilistic method

**DOI:** 10.1371/journal.pone.0223785

**Published:** 2019-10-29

**Authors:** Matthieu Doyen, Di Ge, Alain Beuchée, Guy Carrault, Alfredo I. Hernández

**Affiliations:** Univ Rennes, CHU Rennes, Inserm, LTSI - UMR 1099, F-35000 Rennes, France; Universidad de Zaragoza, SPAIN

## Abstract

Robust, real-time event detection from physiological signals acquired during long-term ambulatory monitoring still represents a major challenge for highly-artifacted signals. In this paper, we propose an original and generic multi-feature probabilistic detector (MFPD) and apply it to real-time QRS complex detection under noisy conditions. The MFPD method calculates a binary Bayesian probability for each derived feature and makes a centralized fusion, using the Kullback-Leibler divergence. The method is evaluated on two ECG databases: 1) the MIT-BIH arrhythmia database from Physionet containing clean ECG signals, 2) a benchmark noisy database created by adding noise recordings of the MIT-BIH noise stress test database, also from Physionet, to the MIT-BIH arrhythmia database. Results are compared with a well-known wavelet-based detector, and two recently published detectors: one based on spatiotemporal characteristic of the QRS complex and the second, as the MFDP, based on feature calculations from the University of New South Wales detector (UNSW). For both benchmark Physionet databases, the proposed MFPD method achieves the lowest standard deviation in sensitivity and positive predictivity (+P) despite its online algorithm architecture. While the statistics are comparable for low-to mildly artifactual ECG signals, the MFPD outperforms reference methods for artifacted ECG with low SNR levels reaching 87.48 ± 14.21% in sensitivity and 89.39 ± 14.67% in +P as compared to 88.30 ± 17.66% and 86.06 ± 19.67% respectively from UNSW, the best performing reference method. With demonstrations on the extensively studied QRS detection problem, we consider that the proposed generic structure of the multi-feature probabilistic detector should offer promising perspectives for long-term monitoring applications for highly-artifacted signals.

## 1 Introduction

Event detections from physiological signals are often faced with important noise perturbations, especially in clinical monitoring context. Main strategies are focused on finding an efficient feature reliable in most cases. Generally, these methods such as [1] get interesting results under low- to mid-level noise conditions, but performances decrease significantly with the signal-to-noise ratio (SNR) diminution or with a change in the noise type since all features have vulnerabilities to specific distortions. To circumvent this weakness, multi-feature detectors were proposed [2] but the decentralized fusion method does not fully exploit feature informations using statistical learning strategies. The main objective of this paper is to propose a generic event detection method with centralized fusion, in which the final decision is made by a weighted sum of posterior detection probabilities derived from each feature’s statistical properties. The power of the method is illustrated by its application to real-time QRS complex detection from electrocardiogram (ECG) signals.

QRS complex is the most prominent deflection in ECG signal and corresponds to the electrical depolarization of ventricles. The detection is often the first analysis performed on ECG signal processing, in order to estimate basic cardiac markers, such as heart rate or to perform further ECG segmentation and analysis. The QRS complex detection has been investigated for many decades [1] and yet remains a challenge [3] as an event detection problem from physiological signals. Many different methods have been proposed and a number of review publications have been dedicated to this subject [4] [5]. The main proposed methods are based on filtering and non linear transformations [1], fuzzy hybrid neural networks [6], S-Transform [7] or wavelet analysis [8–11]. Although these QRS detection methods perform well in low- to mid-level noise conditions, their applications on long-term periods of ECG recordings under noisy conditions such as in ambulatory care and intensive care units still pose a significant challenge as evidenced by the large number of RR correction methods reported in [12]. Indeed, these ECG recordings are often prone to episodes of strong signal non-stationarity, sudden modifications of beat morphologies and most importantly the presence of several types of noise (baseline drift, saturation, power-line pickup, muscular contractions and motion artifacts [13]). Recently in [14], the authors explained that QRS detection has not been completely assessed in terms of robustness to noise. As a consequence, recent publications [15] [16] [17] and a recent PhysioNet challenge [3] have been focused on the specific problem of robust QRS detection. Furthermore, the emergence of wearable cardiac monitors, with a limited number of leads [18] [19] for long-term daily-life recordings [20] further revives the research interests on robust QRS detection for low-quality electrocardiograms.

In our previous works, we have proposed different methods to improve the robustness of QRS detection, through multisensor fusion [2], adaptive selection of QRS detectors as a function of the signal context [21] or through optimal detector parameter configuration, using evolutionary methods [22] [23]. More recently, we revisited this optimization process in order to identify optimal parameter configurations with respect to changes in signal noise [24].

In this paper, we propose and evaluate a novel, generic event detector, that provides improved robustness through the probabilistic combination of a set of signal features. Section 2 presents the general architecture of the proposed Multi-Feature Probabilistic Detector (MFPD) and a specific implementation adapted to robust QRS detection. Section 4 evaluates its performances on two ECG databases: 1) the benchmark MIT-BIH [25] to validate the proposed method on clean ECG signals with consensus annotations, 2) the benchmark noisy database (created by adding noise recordings to the MIT-BIH) with known artifact types and levels, to validate the proposed method on highly artifactual ECG signals including various artifacts.

## 2 Methods

### 2.1 General architecture of the detector

**Fig 1:**
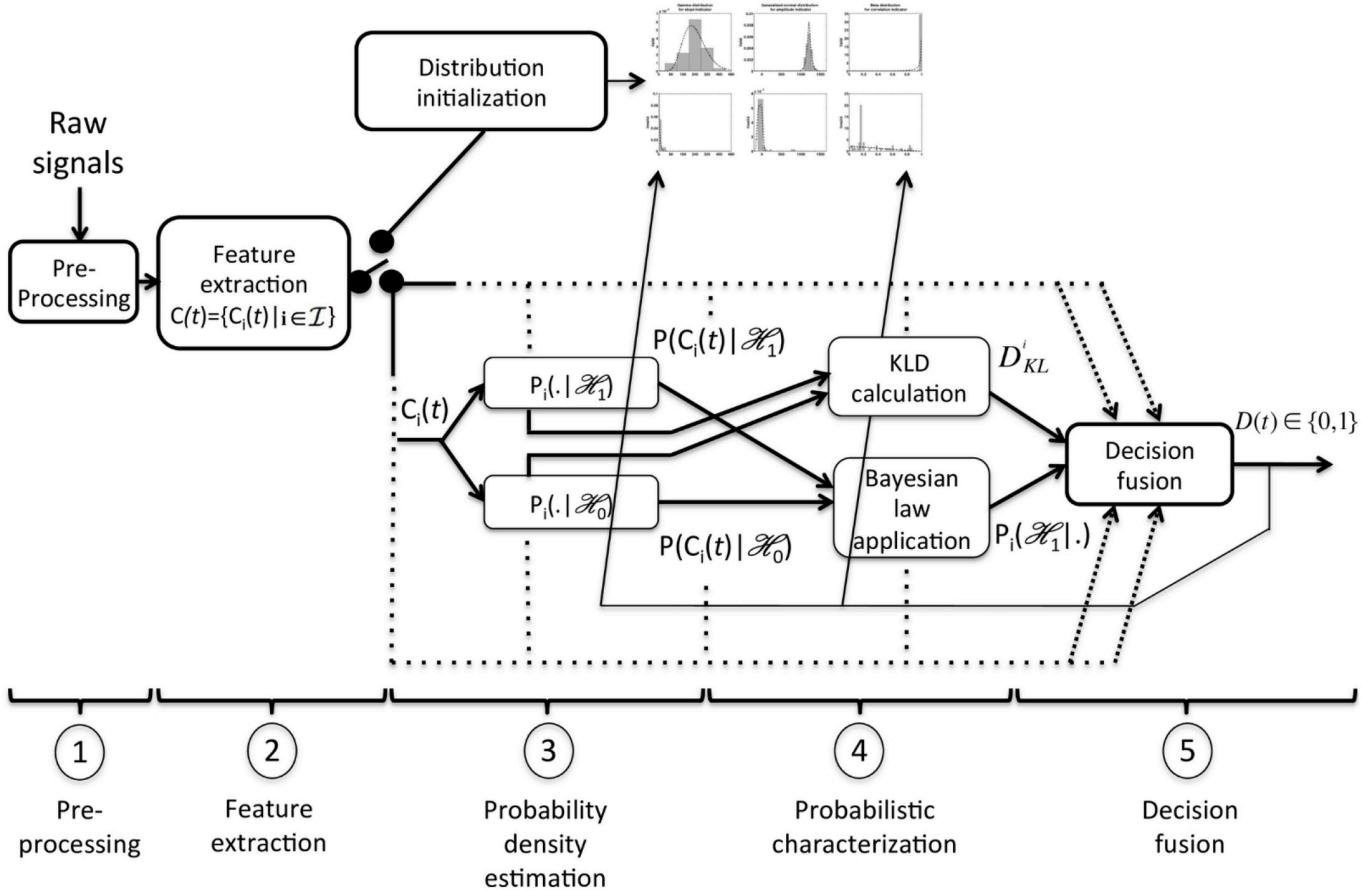
Global architecture of the MFPD. In the first two steps, data coming from ECG are filtered then converted into features *C*_*i*_(*t*). $P_{i}(.|H_{1})$ and $P_{i}(.|H_{0})$ are parametric probability models of feature i, representing valid and invalid detections.

The general architecture of the MFPD depicted in Fig 1 is based on the following steps:

1. Pre-processing: Raw signals are processed in order to improve the SNR and to pre-select potential candidates (to be validated by the detector) of the events of interest at instants *t*.
2. Feature extraction: For every event candidate selected at instant *t*, a vector $C\left(t\right)=\{C_{i}\left(t\right)|i\in I\}$ is created, where I is a set of complementary features extracted from the preprocessed signals.
3. Probability density estimation: The probability density functions (pdf), noted as $P_{i}(C_{i}(t);\Theta_{i0/1},H_{0/1})$ are used to model feature *i* on the observed candidate *C*(*t*), with the two hypotheses:
  $$\begin{array}{c} H_{0}:D(t)=0,\\ H_{1}:D(t)=1, \end{array}$$
  where *D*(*t*) is the final detection decision (*D*(*t*) = 1 for detection and *D*(*t*) = 0 otherwise) and Θ_*i*0/1_ the parameter set for each hypothesis. Note that for each feature, the two pdf belong to the same distribution family, whose parameters Θ_*i*0/1_ are initialized at the beginning of the recording and updated throughout the detection process (see Section 2.2.3 for more details).
4. Probabilistic characterization: The posterior probability $P_{i}(H_{1}|C_{i}(t))$ is calculated by applying the Bayes law. Moreover, the Kullback-Leibler divergence (KLD) between each pdf pair characterizing feature *i*, $D_{\text{KL}}^{i}$, is calculated.
5. Decision fusion: The posterior probabilities, weighted by their respective KLD, are combined to build a binary decision *D*(*t*) on whether candidate *C*(*t*) is a valid event (*D*(*t*) = 1) or not (*D*(*t*) = 0). According to the decision for the current candidate, distribution parameters are updated to complete the real-time learning process.

### 2.2 Real-time QRS detection implementation

**Fig 2:**
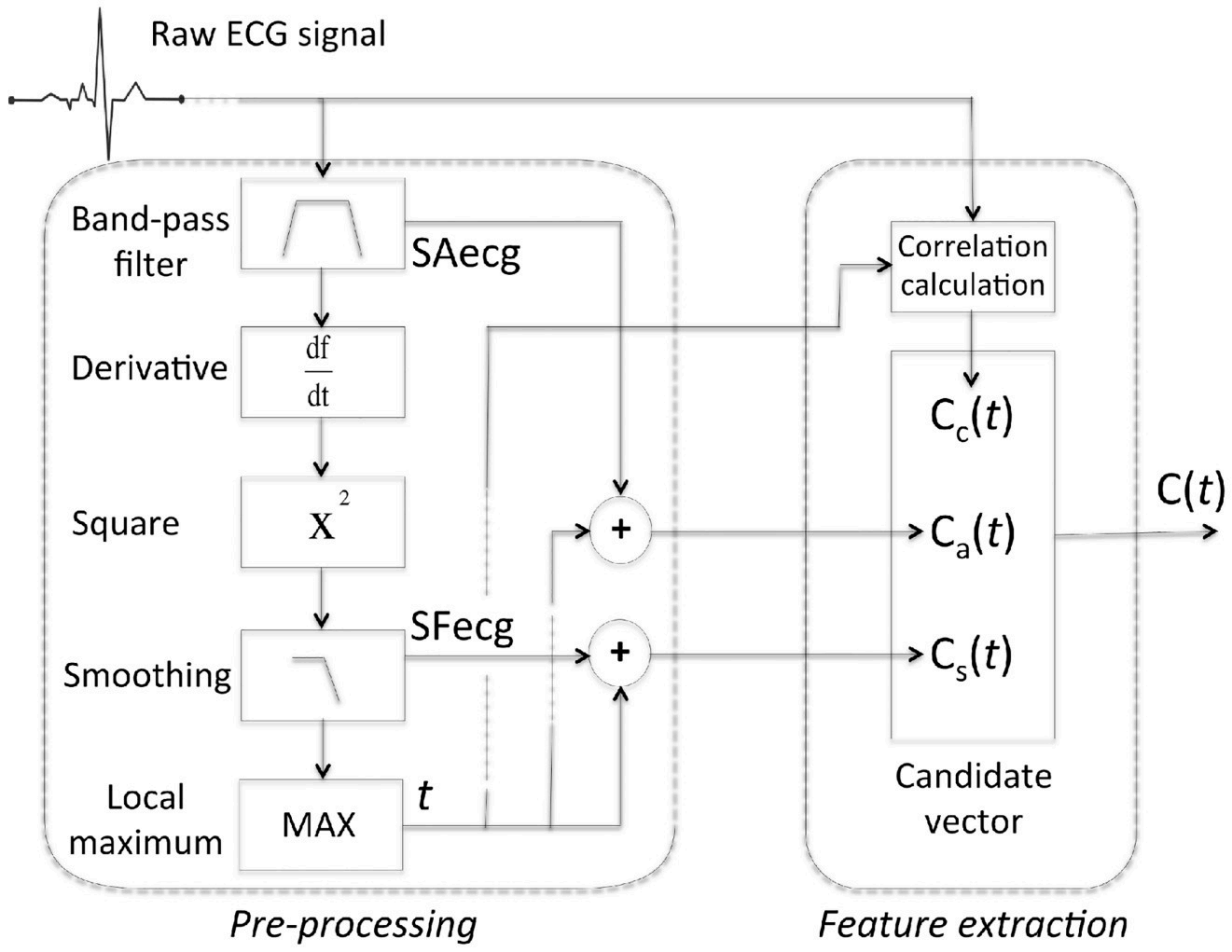
Specification of signal pre-processing and feature extraction (steps 1 and 2 in Fig 1). For the application of robust QRS detection, a set of 3 features $C\left(t\right)=\{C_{i}\left(t\right),i\in I\},I=\left\{s,a,c\right\}$ is extracted.

In this section, we detail the realization of the above-mentioned MFPD, adapted for real-time detection of QRS complexes. From the generic approach of Fig 1, the specific adaptions to QRS complex detection include mainly the pre-processing (step 1) and the feature extraction (step 2), they are depicted in Fig 2.

#### 2.2.1 Pre-processing

As in many other QRS detection methods, the first step consists in applying to the raw ECG signal different transformations. Typically, a band-pass filter, a derivative filter, a non-linear transformation and a final smoothing filter are applied. We adopt here the pre-processing method used in [1, 26]: Fig 2 represents a diagram of these steps. In the following, the band-pass filtered ECG signal, computed using finite impulse response (FIR) low pass (*f*_*cutoff*_ = 19 Hz, order = 256) and high pass (*f*_*cutoff*_ = 8 Hz, order = 256) filters, will be denoted SAecg; and the output of a squared transformation followed by a derivative FIR filter (*f*_*cutoff*_ = 30 Hz, order = 129) then a smoothing filter (length = 101 ms) on signal SAecg will be denoted SFecg (cf Fig 2). FIR filters were designed using Remez exchange algorithm. Each local maximum detected at an instant *t* on SFecg is considered as a potential QRS candidate.

#### 2.2.2 Feature extraction

In this paper, a set of 3 features $C\left(t\right)=\{C_{i}\left(t\right),i\in I\},I=\left\{s,a,c\right\}$ is extracted for each QRS candidate. These features are the input to the probability density estimation step (step 3 in Section 2.1 and Fig 1). The squared slope (s), the raw amplitude (a) and the absolute correlation with a beat template (c) constitute the most common features used in the literature for QRS detection. In the current study, we prove the concept of decision fusion with adaptive weights to track the relative importance of each feature while the optimization of feature selection lies beyond the scope of our investigation.

#### 2.2.3 Feature probability density

1. Feature probability models: in the proposed QRS detection application, each candidate is characterized by a set of 3 features I={s,a,c}. The probability distribution function (pdf) of features are chosen with several conditions: 1) distributions should have the same support as the calculated feature, e.g. (0, ∞) for the squared slope, (−∞, + ∞) for the amplitude and [0, 1] for the absolute correlation, 2) pdf parameter estimations should be easy to calculate, with low complexity in the updating methods, 3) the distance measures on these pdfs should also be easy to compute and tractable, without numerical integrations. In this paper, we implemented three different pdfs: (i) a Beta distribution with closed support, (ii) a Gamma distribution with one-sided open support, and (iii) a generalized normal distribution (GND) with two-side open support. Thus, pdf parameter update and distance calculation methods in this paper can be generalized to include most continuous features in the future.
  - The squared slope of the peak (*s*) is the value of SFecg signal at instant *t*. This feature is represented with the Gamma distribution with two degrees of freedom:
    $$\begin{array}{c} P_{s}\left(x;k,\theta \right)=\frac{x^{k-1}e^{-\frac{x}{\theta }}}{\Gamma \left(k\right)\theta^{k}}\;{1\;\;1}_{x>0}, \end{array}$$ (1)
    where $k\in R^{+}$ is the shape parameter, and $\theta \in R^{+}$ the scale parameter. The indicator function ${1\;\;1}_{x>0}$ typically limits the function support to $R^{+}$.
  - The peak amplitude (*a*) is the value of SAecg signal at instant *t*. We characterized it using the GND defined as:
    $$\begin{array}{c} P_{a}\left(x;\alpha ;\beta ;\mu \right)=\frac{\beta }{2\alpha \Gamma (1/\beta )}\;e^{-{\left(\frac{\left|x-\mu \right|}{\alpha }\right)}^{\beta }} \end{array}$$ (2)
    where $\Gamma \left(t\right)=\int_{0}^{\infty }\;\;\;x^{t-1}e^{-x}\text{d}x$ is the gamma function and μ∈R the position parameter, $\alpha \;\in \;R^{+}$ the scale parameter and $\beta \;\;\in \;R^{+}$ the shape parameter. Note that both positive and negative peak values can be fitted with the GND model.
  - The absolute Bravais-Pearson correlation (*c*) is calculated between the candidate peak (represented by 50 ms of raw ECG signal centered 20 ms before the peak) at instant *t* and an adaptive template. The template duration was chosen in order to extract mainly the information around the peak, where the information is most characteristic of the QRS complex in our opinion. With a longer template duration, QRS complexes affected by noise can obtain a lower correlation, this weakness is less present with a short template duration. With an even shorter duration, high-frequency artifacts can be very similar to QRS complexes. In order to model this feature, we have chosen the Beta distribution, defined as:
    $$\begin{array}{c} P_{c}\left(x;\alpha ,\beta \right)=\frac{\Gamma (\alpha +\beta )}{\Gamma (\alpha )\Gamma (\beta )}\;x^{\alpha -1}{\left(1-x\right)}^{\beta -1}\;1\;\;1_{\left[0,1\right]}\left(x\right). \end{array}$$ (3)
    This can also be considered as a special case of the Dirichlet distribution, with two positive shape parameters *α* and *β*. Parameters are estimated using maximum a posteriori (MAP) method, further details are reported in section 4.2.
  Note again that the same pdf family is proposed for both $H_{0}$ and $H_{1}$ considering the numerical tractability of the KLD calculations (see discussion in 2.2.4).
2. Initialization: As in step 3) in section 2.1, the model parameters—{*k*, *θ*} for the Gamma distribution, {*α*, *β*, *μ*} for the GND, and {*α*, *β*} for the Beta distribution—are initialized during the heating-up period (cf block *distribution initialization* in Fig 1) using the Pan-Tompkins detector [1] for the 40 first validated QRS detections (note that these detections are considered in the performance evaluation step). At the end of the heating-up period, all QRS candidates are labelled as either validated ($H_{1}$) or invalidated ($H_{0}$) and the model parameters are estimated for each distribution, using the maximum likelihood estimator (MLE) for the *s* and *a* feature, and MAP estimator for the *c* feature. Beat templates (for both $H_{1}$ and $H_{0}$) are also initialized (aligned and averaged) using the validated and invalidated candidates of the heating-up.
3. Learning: Once initialized, the MFPD detector shifts to the decision fusion mode (step 3-5 in section 2.1 and Fig 1) whose final detection results (of step 5) feed the pdf parameter updating (step 3) in the same manner as during the initialization. KLDs are updated as a direct consequence (step 4). In a similar manner, the beat templates are updated after each decision using 80% of the previous template and 20% of the current candidate. If no detection occurs during 3.5 sec, all parameters are reset and a new initialization starts.

#### 2.2.4 Probabilistic characterization

Based on the pdf model for each feature in 2.2.3, two probabilistic markers are calculated for each feature: the posterior probability and the KLD. The posterior probability of validating $H_{1}$ for feature *C*_*i*_(*t*) is given by

$$\begin{array}{c} P_{i}\left(H_{1}|C_{i}\left(t\right)\right)=\frac{P_{i}\left(C_{i}\left(t\right)|H_{1}\right)\;P_{i}\left(H_{1}\right)}{P_{i}\left(C_{i}\left(t\right)|H_{1}\right)P_{i}\left(H_{1}\right)+P_{i}\left(C_{i}\left(t\right)|H_{0}\right)P_{i}\left(H_{0}\right)} \end{array}$$ (4)

using the Bayes rule. A binary decision can typically be made by thresholding this probability as in most single feature-based QRS detectors.

We propose in this paper a centralized fusion by making a weighted sum of these posterior probabilities. The key here is to update dynamically a metric to measure the pertinence of each feature, or the power of separating two classes in the detection context by measuring the distance between the two antagonist pdfs. The KLD is such a non-negative measure defined by:

$$\begin{array}{c} D_{\text{KL}}\left(p\|q\right)=\int_{-\infty }^{\infty }p\left(x\right)\;\text{log}\frac{p(x)}{q(x)}\;\text{d}x. \end{array}$$ (5)

It is particularly well-suited to assess the distance between the distribution pair $P_{i}(C_{i}(t);\Theta_{i0/1};H_{0/1})$. Analytic expressions can be found in the literature in the case of Beta [27] and Gamma distributions [28]. However for the GND case and to our best knowledge, no analytic expression can be found in the general case when *μ*_*p*_ ≠ *μ*_*q*_. One theoretical contribution of this paper is to efficiently calculate the KLD between two GND distributions in the case of general settings. While detailed derivation is reported in the appendix B, here we give a summary of the main results:

1. Analytic expressions for $\beta_{q}\;\in \;N^{+}\;\cup \;\left\{0\right\}$ have been derived;
2. Eq (5) is monotonously increasing with *β*_*q*_;
3. The computational complexity requires 2 × (*β*_*q*_ + 1) gamma function evaluations.

Thus without numerical integration of Eq (5), we are able to obtain a close approximation of the KLD value for all $\beta_{q}\in R^{+}$. Note that the other parameters (*α*_*p*_, *α*_*q*_, *β*_*p*_, *μ*_*p*_, *μ*_*q*_) have no effect on the calculation complexity.

Finally, and without loss of generality, the KLD is one of the *f*-divergence functions that measures the difference between two probability distributions. They are non-negative, monotonous and jointly convex. For example, the reverse KLD $D_{\text{KL}}^{R}\left(p\|q\right)=D_{\text{KL}}\left(q\|p\right)$ is another *f*-divergence and can be easily calculated by reversing the role of the two distributions. However, it is less evident to derive tractable numerical methods for the Hellinger distance, or the *χ*^2^-divergence for the exponential pdf family.

#### 2.2.5 Decision fusion

Based on the probabilistic markers, the following decision rule is applied

$$\begin{array}{l} D(t)=1\;\text{if}\;\text{and}\;\text{only}\;\text{if}\\ \sum_{i\in I}\frac{{\bar{D}}_{\text{KL}}^{i}.P_{i}(H_{1}|C_{i}(t))}{\sum_{j\in I}{\bar{D}}_{\text{KL}}^{j}}>\lambda \end{array}$$ (6)

where λ is the decision threshold. The modified ${\bar{D}}_{\text{KL}}^{i}$ is calculated by letting $i^{*}=argmax\left\{D_{KL}^{i}\right\}$ and:

$$\begin{array}{c} {\bar{D}}_{KL}^{i}=\{\begin{array}{c} min\{D_{KL}^{i},2\sum_{h\neq i^{*}}D_{KL}^{h}\},\;i=i^{*}\\ D_{KL}^{i},\;i\neq i^{*} \end{array} \end{array}$$ (7)

Such that the most significant KLD should not exceed 2/3 after normalization. Intuitively, the decision rule represents the sum of all posterior probabilities, weighted by their normalized KLD, such that features that are better separated in distributions (between $H_{0}$ and $H_{1}$) have more weight in the final decision making.

## 3 Performance evaluation for QRS detection

### 3.1 Databases

Two databases were used:

- Benchmark database: we first performed a comparative study using the first lead of the MIT-BIH Arrhythmia Database with consensus annotations to show the detection performances on clean ECG signals even though the main objective of the paper is to evaluate detector’s robustness under artifact conditions.
- Benchmark noisy database: we then created a benchmark simulated database by adding to the MIT-BIH Arrhythmia Database three noise sources (baseline wander, muscle and electrode motion artifact) extracted from the MIT-BIH noise stress test database. Noises were recorded from physically active volunteers [25] and with SNR levels from −6dB to 24dB by 6dB increments. It is composed of 864 noisy signals for which the reference annotations are simply copies of those for the MIT-BIH Arrhythmia Database. The purpose of this database test is to provide a ground truth for detection performance comparison with different levels and types of noises. A quick access to this benchmark database is available at: https://github.com/dge996/MIT_NoiseStress/ though it can also be constructed using the method described in [25].

We choose to report results on two databases to prove the design concept of the MFPD robustness for clean and noisy databases with consensus annotations. For the second one, both artifact type and level classification annotations are provided, they allow differentiated comparisons.

### 3.2 Comparison methods

As in recent publications [29] [15], performance of the proposed MFPD was compared with the following state of the art QRS detection methods:

- University of New South Wales detector (UNSW): a feature-based (ECG amplitude and derivative) detector, with adaptive thresholding, suitable for both clinical and poorer quality tele-health ECG [15].
- Spatiotemporal Characteristics Detector (SCD): a simple and robust realtime QRS detection algorithm based on spatiotemporal characteristics [30], with state-of-the-art performances reported for noisy signals.
- Wavelet-Based Detector (WBD): a wavelet-based QRS detector [31], with implementation provided in the ECG-toolkit [32] [33].

WBD was chosen because it is a reference in QRS detection. The other two methods were selected because their objectives are the same as MFDP, which is not the case for many detectors from the literature focused on clean ECG signals. SCD is described by their authors as robust and real-time, UNSW detector is described as suitable for poorer quality ECG signals. Moreover, both were recently published (2016) with open access toolbox. We underline however that contrary to UNSW and WBD methods, the MFPD is an online algorithm designed for monitoring applications which forbids typically bi-directional filtering and beat by beat corrections in post-treatment. We thus further included detection delay statistics, that cannot be obtained for classical detectors.

### 3.3 Performance criterion

In this study, we used the bxb program (as part of the WFDB applications available on physionet) to obtain the beat-by-beat performance statistics for QRS complex detection according to the American national standard for ambulatory ECG analyzers (ANSI/AAMI EC38). We report for the two databases the total number of TP, FP and FN as well as the sensitivity (Se) and positive predictivity (+P) metrics calculated per record and a mean and standard deviation alongside the overall result. As in recent publications [24] [34], we chose a match window of 50 ms. Only one detection is considered as true positive (TP) inside the match window centered at each QRS annotation while the rest are considered as false positive (FP) detections. A false negative (FN) is counted when no detection is found inside the match window. Due to their direct influences on heart rate variability analysis, we also report the mean and standard deviation of *jitters*: the difference in time between a TP and its associated annotation. To easily observe the increase in detection performance as a whole, the detection error rate DER = (FN+FP)/(TP+FN) is also reported. Furthermore, computational complexity and detection delay of the MFPD are also given since they are key implementation issues in realtime monitoring applications.

## 4 Results

In addition to the global performance evaluation and comparison summarized in 4.4, we also provide here some intermediate results to illustrate the multi-feature complementarity in 4.1, their distribution estimation results in 4.2, and the importance of KLD weighting in the centralized decision making in 4.3.

### 4.1 Multi-feature complementarity

**Fig 3:**
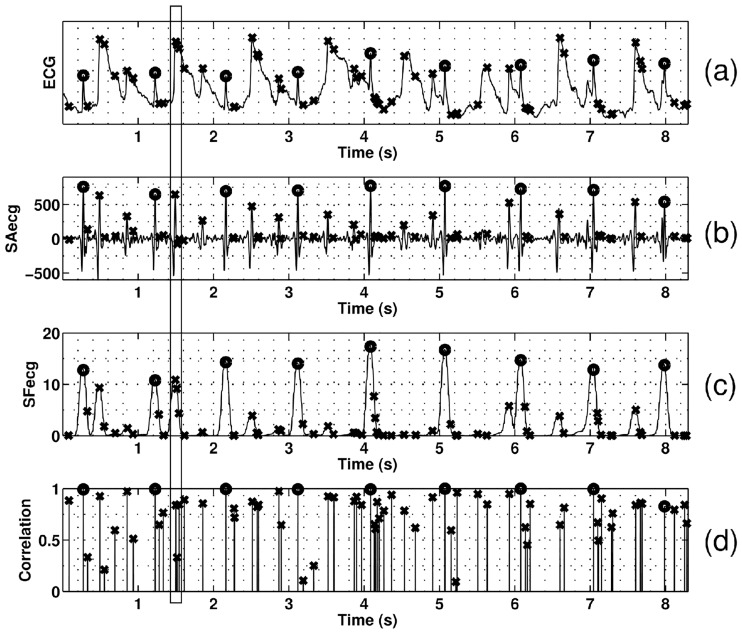
Illustration of the multi-feature complementarity in fusion decision. a) Raw ECG segment from record MIT-101 (SNR = 6 dB with electrode noise) b), c), d) represents the feature *a*, *s*, *c* respectively. The × and ⚬ indicate invalidated and validated QRS respectively by the fusion.

Fig 3 shows an example of the processed ECG signals and the features extracted in this paper. Panel (a) shows an ECG segment from record MIT-101, with added electrode movement noise at 6 dB. Panels (b) (c) and (d) represent, respectively, the SAecg (feature *a*), the SFecg (feature *s*) signals and the Bravais-Pearson correlation (feature *c*) related to each QRS complex candidates in panel (a). Candidates with the × symbol are not validated, while those with the ⚬ symbol are validated as QRS by the MFPD method. In this example, all validated detections were TP and all invalidated candidates were true negatives (TN), not belonging to any annotation’s match window. The vertical box pinpoints a segment that, if analyzed individually with thresholding, would have produced a false positive. The weighted fusion by Eq (6) however takes the opposite decision. This example shows the power of the multi-feature complementarity in such complex signal context.

### 4.2 Distribution estimation

**Fig 4:**
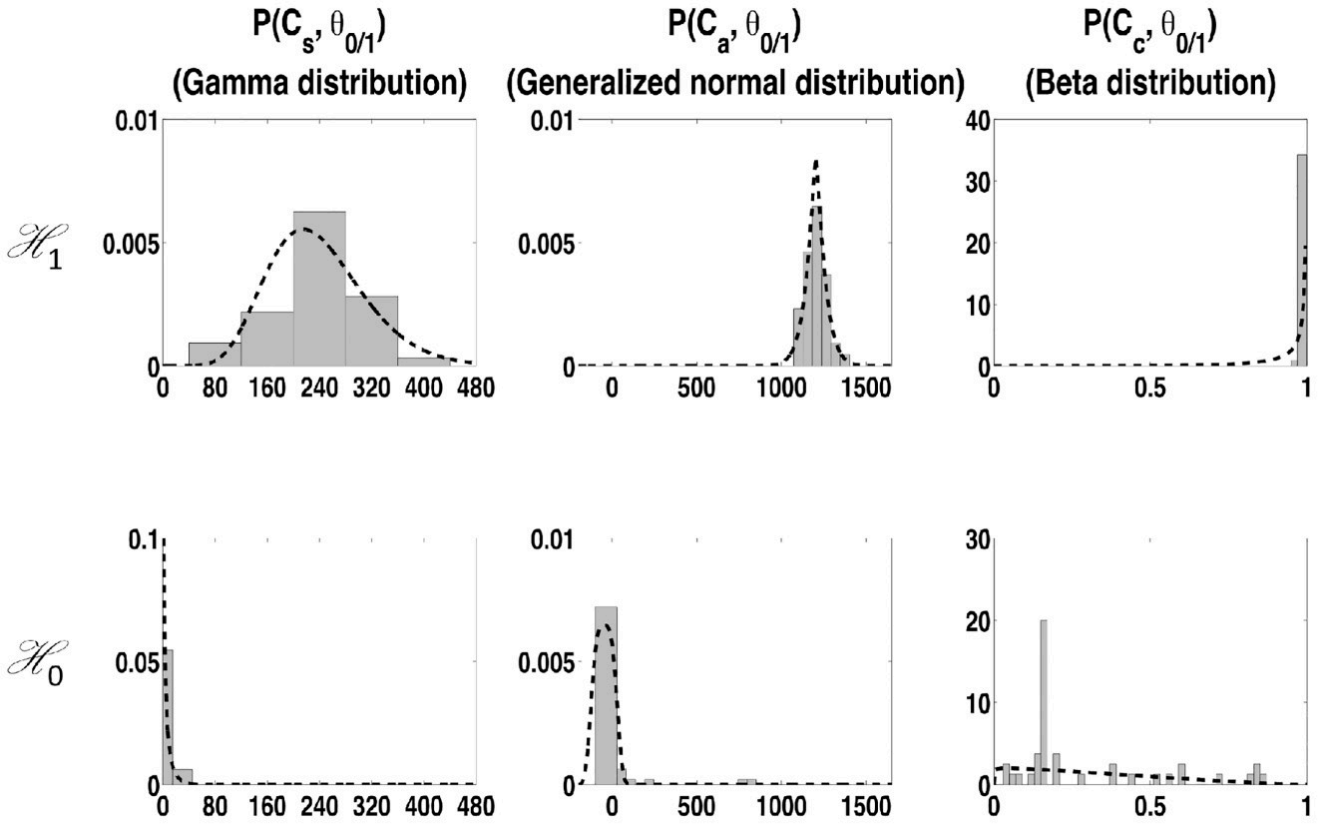
Estimated distributions (dashed line) vs normalized histograms (vertical boxes) for the three features and for both $H_{0}$ and $H_{1}$. Record MIT-101 is used with added baseline noise (SNR = 12dB).

Estimated parametric distributions (in dashed line) and normalized histogram (in vertical boxes) for each feature are illustrated in Fig 4. The x-axis stands for the dimensionless feature values while y-axis the probability density (also dimensionless). Generally speaking, the pdf type associated with updated parameters can reasonably fit the feature histograms. Kolmogorov–Smirnov tests were realized at each pdf parameter update, with the current candidates history. Of the 10678 tests performed for the MIT-101 signal, all were positive at the 0.05 significance level.

For mildly-artifacted ECG signals, due to the stability of the QRS complex morphology, the correlation features *C*_*c*_(*t*) can be close to 1 for most valid candidates. Thus large *α* values are estimated for the beta distribution $P_{c}(C_{c}(t);\Theta_{c1},H_{1})$ yielding a rapid convergence towards a dirac-like distribution around 1, and a large $D_{KL}^{c}$ value (cf the net separation in the two distributions of the third column in Fig 4). As a consequence, future candidates must have a very high correlation to be validated, giving a decision with a high +P, but with a low sensitivity in the case of sudden noise artifacts or even mild morphology change. In order to obtain a better trade-off between +P and sensitivity, we propose to limit the estimated *α* parameter of the beta distribution by imposing a conjugate prior law:

$$\begin{array}{c} P\left(\alpha ,\beta \right)\propto B{\left(\alpha ,\beta \right)}^{K}e^{-a\alpha }e^{-b\beta } \end{array}$$

for $K,a,b\in N^{+}$. The exp^−*aα*^ term indeed forbids large values in estimating *α* to control the distribution shape of $H_{1}$. Numerical implementation is detailed in appendix A for the MAP estimator.

### 4.3 KLD weighting

**Fig 5:**
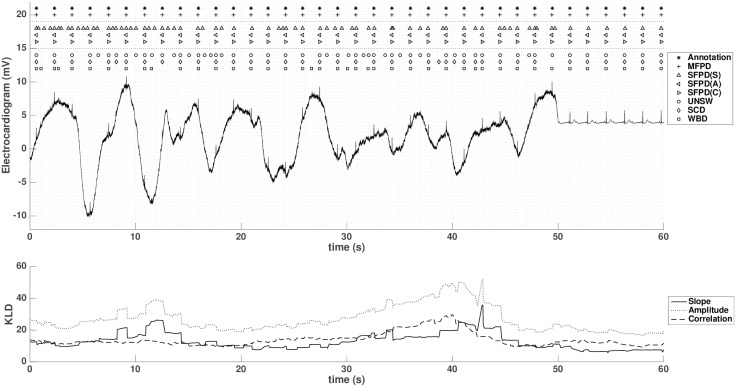
KLD variations for successive QRS candidates in 60 s from record MIT-231 with baseline noise and -6dB SNR. Upper panel: raw ECG signal with annotations (marked by *), MFPD fusion results (+), single feature (SFPD) results (△, ⊲ and ⊳), and results of the reference methods (⚬ for UNSW, ◊ for SCD and □ for WBD). Lower panel: KLD evolution for three features in the same period.

In this section, we show the importance of the KLD measure in comparison with both Single Featured Probabilistic Detector (SFPD) and reference methods in Fig 5. SFPD is implemented as MFPD but with only one feature (*s*, *a* or *c*). Note that the evolution of KLD values during the successive (in)-validation process is a direct consequence of the parameter-fitting process since Eq (5) is a function of the updated pdf parameters (cf [27, 28], Appendix B). Indeed, the relative importance of the KLD of the *a*-feature during artifactual period suggests a better separability between the two antagonist distributions ($H_{0}$ vs $H_{1}$) and consequently highlights the decision from the *a*-feature in the centralized fusion (see Eq (6)). Interestingly, the KLD values tend to approach each other once the artifact period ends (near 50s), from which point all feature decisions participate more equally in the fusion. On the other hand, that the SFPD using the *s*-feature performs the worst (with multiple FP detections) is inline with its lowest KLD measure, or the smallest distances between the related $H_{0}$ and $H_{1}$ pdfs. Therefore, depending on the noise onset, the KLD measure of the distribution distances evolve dynamically to quantify the pertinence of each feature and to adapt their relative weights in the decision fusion. The power of the proposed method lies on this engineered flexibility since KLD are not learned from a particular database, but from the past observations of the features labelled by the detection results.

Furthermore, the detection statistics of the whole record MIT-231 are given in Fig 5: the MFPD outperforms the SCD method in both sensitivity and +P, has lower sensitivity than UNSW and WBD but highest in +P (see Table 1), which is consistant with the illustration of the 60s segment detection results in Fig 5.

**Table 1:** Performances of the whole record MIT-231 of Fig 5.

record MIT-231 with baseline noise (−6dB)						
	# TP	#FN	#FP	Se (%)	+P(%)	DER(%)
**MFPD**	1468	103	63	93.44	95.89	10.57
**UNSW**	1524	47	170	97.01	89.96	12.81
**SCD**	1038	533	74	66.07	93.34	38.63
**WBD**	1510	61	223	96.12	87.13	18.08

### 4.4 Performance evaluation

#### 4.4.1 Benchmark MIT-BIH arrhythmia database

In Table 2, we first present the performance comparison on the MIT-BIH database, containing mainly clean ECG recordings, and high Se and +P results for all tested methods. We simply note that the MFPD has the lowest standard deviation for both Se and +P statistics, a phenomenon also observed in the next tests to illustrate its stability. On the whole, the results indicate a trade-off in favor of the +P as compared with the reference methods while the overall score DER also shows high detection accuracy in both mean and standard deviation terms. To illustrate the method’s inter-subject stability, 2.08% of signals had a DER higher than 60% with MFPD versus 6.25% for UNSW, 2.08% for SCD and 8.33% for WBD. This test validates the MFPD on a clean ECG database with consensus annotations and peer methods.

**Table 2:** Performance comparison on the benchmark MIT-BIH arrhythmia database.

Benchmark MIT-BIH DB							
	# TP	#FN	#FP	Se (%)	+P(%)	DER(%)	Jitter (ms)
**MFPD**	101799	7695	3195	93.16±11.46	96.81±9.03	9.86 ± 18.96	7.91±4.62
**UNSW**	103010	6484	6439	94.01±16.44	94.01±16.45	11.97 ± 32.86	7.04±4.77
**SCD**	104942	4552	4631	95.88±13.62	95.79±13.60	8.32 ± 27.20	5.46±3.69
**WBD**	103125	6369	5684	93.88±13.77	94.33±13.77	11.77 ± 27.45	3.08±3.33

Performances on the benchmark MIT-BIH arrhythmia database including: total number of TP, FN, FP, Se and +P (mean and standard deviation in %) and detection jitter (mean and std in ms) for 48 recordings.

#### 4.4.2 MIT-BIH noise stress database

**Fig 6:**
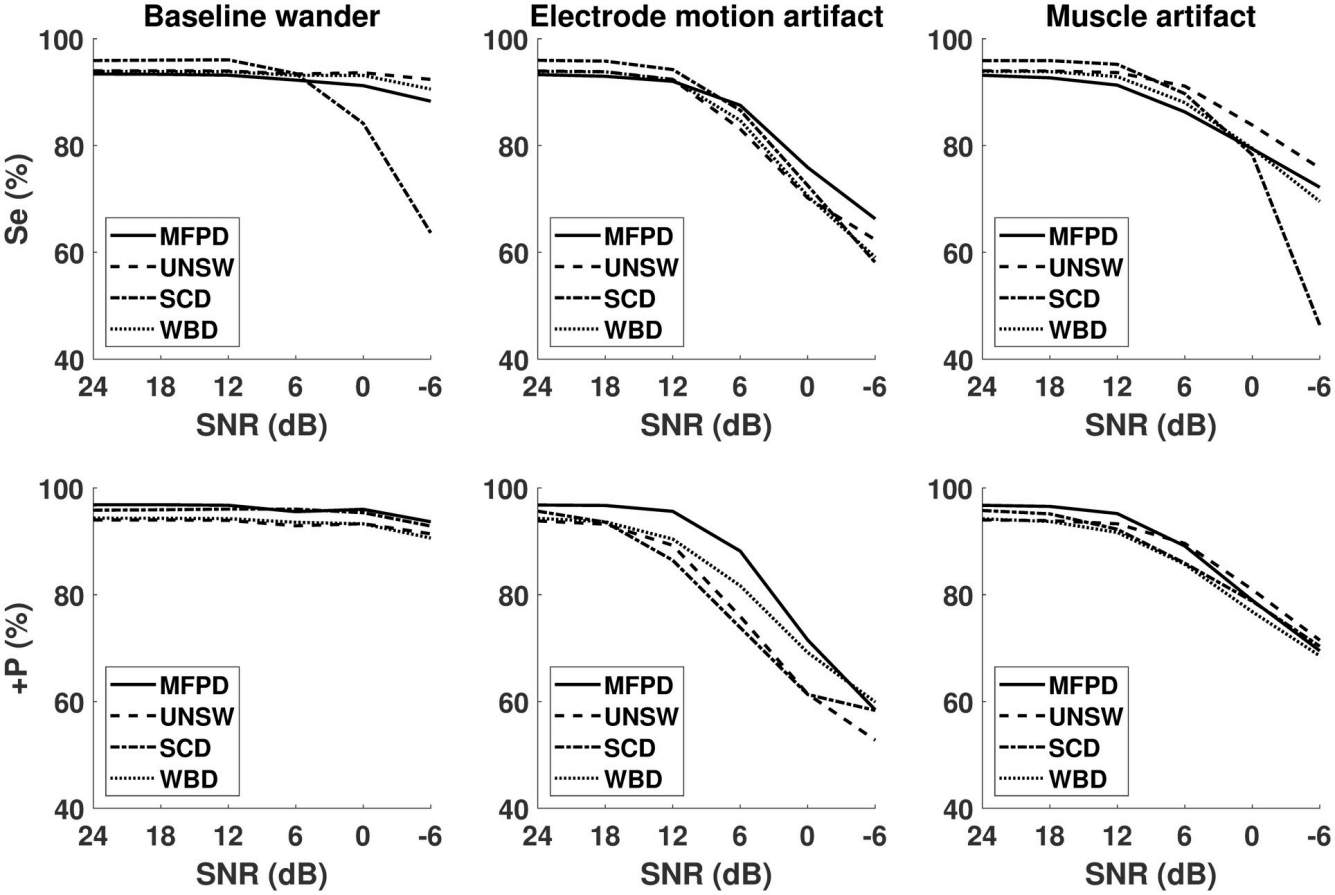
Se and +P (in %) of MFPD, UNSW, SCD and WBD on the benchmark noisy database, with different noise types and SNR levels.

A second performance analysis was done using the MIT-BIH noise stress database. Fig 6 compares Se and +P (in %) for different noise types. Firstly, for +P the MFPD outperforms almost all reference methods except in the case of muscle artifact with SNR lower than 0dB. As for Se, the MFPD has slightly lower performances in the high SNR region for all artifact types, but higher performances in the low SNR regions. In general the MFPD has better Se scores in comparison with the SCD and WBD method as reported by Table 3. The UNSW, on the other hand, has shown comparable performances in most cases, but suffers from FP detections in the presence of electrode motion artifacts. The MFPD also achieves the lowest overall DER score in terms of mean and standard deviation. To illustrate the method’s inter-subject stability, 11.81% of signals had a DER higher than 60% with MFPD versus 16.78% for UNSW, 17.82% for SCD and 16.90% for WBD. These results demonstrate the MFPD viability and highlight its efficiency in noisy context.

**Table 3:** Performance comparison on the benchmark noisy database.

Benchmark Noisy DB							
	# TP	#FN	#FP	Se (%)	+P (%)	DER (%)	Jitter (ms)
**MFPD**	1723	247	208	87.48±14.21	89.39±14.67	23.45 ± 28.46	8.15±4.24
**UNSW**	1743	227	287	88.30±17.66	86.06±19.27	26.82 ± 38.47	7.23±4.38
**SCD**	1679	291	257	85.21±18.63	86.64±16.71	28.43 ± 33.54	5.60±3.24
**WBD**	1724	246	250	87.25±16.41	86.68±16.67	26.38 ± 33.09	3.32±3.25

Performances on the benchmark noisy database including: total number of TP, FN, FP (in ×10^3^), Se and +P (mean and standard deviation in %) and detection jitter (mean and std in ms) for all 864 recordings.

Finally, we can notice that performances with baseline wander artifacts are generally higher than with other noise types for all tested methods, which can be attributed to the preprocessing filtering steps that attenuate baseline wanders.

### 4.5 Complexity and realtime detection delay

For the computation complexity, the MFPD algorithm implemented in the C++ language costs 19.32 ± 4.75 s to analyze an hour of ECG signal resampled at 1000 Hz using a MacBook Pro laptop (2017, 3,1 GHz Intel Core i7) without multi-threading or graphic parallel computing. The resampling at 1000 Hz was done because, according to us, this frequency is more representative of actual ECG acquisition devices and easier to interpret for readers.

In realtime monitoring applications, event detection delay is also part of the performance that can be used to trade-off for more accuracy for example. It is different from the jitter reported in Tables 2 and 3 that measures the time distance between annotation and detection. The delay reported in Table 4 measures the time distance between the annotation and the sample time at which the MFPD fusion decision validates the corresponding QRS and is typically influenced by lengths of the filters and the templates to calculate the correlation feature. Table 4 shows that this delay in the monitoring is rather stable across the two databases. These results confirm the feasibility of the MFPD in realtime monitoring of ECG signals, even for multi-lead recordings.

**Table 4:** MFPD detection delay (mean and std in ms).

Detection delay in ms	
MIT-BIH arrhythmia	MIT-BIH noise stress
612±84	648±78

## 5 Discussion

In this paper, a novel, generic and robust detector combining different features extracted from the signal of interest has been proposed. The original aspects of this method concern particularly i) the probabilistic approach with online learning ii) the multi-feature design iii) the centralized fusion method based on KLD.

In the proposed method, the pdf of each feature is patient, device and even experience specific. Parametric probability models are designed with regard to the real-time constraints of our application to avoid the tuning of the number of bins and widths as in histogram based approaches, and the increasing evaluation costs, inherent to variable-bandwith kernel density estimation approaches [35]. Our proposed online learning method requires a small data sample (40 validated candidates) to initialize the probabilistic model for each recording. No manual annotations are needed since the Pan-Tompkins detector [1] results are used for feature extraction and classification labeling ($H_{0}$/$H_{1}$). Prior laws of the Beta distribution for the correlation feature are fixed and not database dependent. Among the wide variety of pdf families, the GND seems well-suited for uncentered features with long tail distributions, but to our best knowledge, no analytic expression of the KLD can be found in the general case. Existing solutions [36] are limited to cases of equal means (see 2.2.4). We proposed in this paper an innovative estimation method of the KLD in the general case. With a reasonable computational cost, it can be used in real-time context.

The proposed MFPD QRS detector has been evaluated using two different databases and compared with two most recent state of the art detectors and one reference detector from the literature. Results show that the features provide complementary information to improve detection performance compared with single featured based QRS detectors (see Fig 5), particularly under noisy conditions. This is essentially due to the fact that different types of noise or uncommon pathological artifacts might influence features in different manners while MFPD makes a centralized decision using the KLD as weight to measure the relative pertinence of each feature. In a way, the MFPD is capable of neglecting features (that yield low KLD between the antagonist distributions) that are mostly corrupted by artefacts. Previously proposed methods based on decentralized fusion [2] and algorithm-switching [21] also prove the relevance of multi-feature approaches. To our knowledge, this is the first real-time QRS detection method integrating such an adaptive, multi-feature, centralized decision fusion. Furthermore, MFPD method is more compact and easy to implement than [2] or [21]. Quantitative comparisons results are particularly encouraging for challenging monitoring situations, in which the heterogeneity and levels of noise may be particularly high. For the two databases under evaluation, the ms-level jitter for all tested methods should be acceptable for further HRV parameter analysis and is thus not regarded as a distinguishing factor for comparison.

Even though this method was implemented for single-lead ECG signals, it can be extended to the multi-lead and multi-source cases. Indeed, further improvements in detection robustness are expected by combining multiple ECG leads, but also by integrating other physiological signals (pulse oximetry, phonocardiography, etc) or other sensors sensitive to noise (accelerometers for movement noise, etc). Future works will be directed towards the extension and evaluation of the method in these multi-channel, multi-source contexts. Finally, in addition to the qualitative results of the selected features’ relevance shown in Fig 3 and in Fig 5 through KLD evolution, integrating the amplitude and derivative features as used by UNSW [15] into the MFPD architecture should lead to higher sensitivity in QRS detection.

## 6 Conclusion

We proposed an original multi-feature probabilistic detector working in real-time and applicable to different physiological signal applications. The method, illustrated on QRS complex detection, has been compared to two latest detectors in the literature, using the MIT-BIH arrhythmia benchmark database and a benchmark noisy database by adding noise recordings [25] to the MIT-BIH database. The proposed MFPD has achieved significant performance improvements on artifacted signals and comparable (in mean) but more stable (in std) performances for low-to mildly artifacted signals. These performance improvements are mainly due to the multi-feature probabilistic model and the KLD-based decision fusion that adaptively adjust the relative contribution of each feature’s decision in real-time.

The following contributions and originalities can be highlighted. The MFPD uses a probabilistic approach with online learning and an original adaptive, multi-feature, centralized decision fusion based on KLD. Its application on QRS detection achieved notable performance gains on artifacted ECG signals in comparison with one classical and two recent methods. The proposed method can also be easily extended to the multi-lead and multi-source cases, or to other physiological event detection applications. Besides the theoretical contribution and experimental validation, this new approach boasts a reasonable computational cost and thus can be embedded into low-power devices offering interesting possibilities in the current context of connected health applications.

## A Application of the Karush-Kuhn-Tucker on the MAP of Beta distribution

### A.1 About the KKT

Consider the non-linear optimization problem: maximize $f\left(x\right),R^{n}\to R$ (the cost function) subject to *m* inequality and *l* equality constraints:

$$\begin{array}{c} g_{i}\left(x\right)\leq 0,i=1,\ldots ,m,\;\text{and}\;h_{j}\left(x\right)=0,j=1,\ldots ,l. \end{array}$$

Suppose further that both *f*(*x*) and the constraint functions *g*_*i*_(*x*), *h*_*j*_(*x*) are continuously differentiable at a point $\tilde{x}$. If $\tilde{x}$ is a local maximum of *f*(*x*) satisfying some regularity conditions, then there exist the KKT multipliers: $\mu_{i}\in R,i=1,\ldots ,m$ and $\lambda_{j}\in R,j=1,\ldots ,l$ such that:

$$\begin{array}{c} \nabla f(\tilde{x})=\sum \mu_{i}\nabla g_{i}\left(\tilde{x}\right)+\sum \lambda_{j}\nabla h_{j}\left(\tilde{x}\right); \end{array}$$ (8)

$$\begin{array}{c} g_{i}\left(\tilde{x}\right)\leq 0,\;\mu_{i}\geq 0,\;\mu_{i}g_{i}\left(\tilde{x}\right)=0\;\text{for}\;\text{all}\;i=1,\ldots ,m \end{array}$$ (9)

$$\begin{array}{c} h_{j}\left(\tilde{x}\right)=0\;\text{for}\;\text{all}\;j=1,\ldots ,l \end{array}$$ (10)

We note that in the particular case of *m* = 0, the KKT conditions are reduced to the Lagrange conditions. If *g*_*i*_ and *h*_*j*_ are affine functions (MAP of beta distribution), then no regularity condition is needed.

### A.2 MAP for the Beta distribution

We derive here the numerical method to calculate the maximum-likelihood estimator (MLE) and MAP estimator given *N* independent samples of the Beta distribution. Recall the Beta density function:

$$\begin{array}{c} P\left(x;\alpha ,\beta \right)=\frac{1}{B(\alpha ,\beta )}x^{\alpha -1}{\left(1-x\right)}^{\beta -1}1_{0\leq x\leq 1} \end{array}$$

where *B*(*α*, *β*) is the normalization constant. For the MLE, the function to maximize is the joint log-likelihood function:

$$\begin{array}{c} f\left(\alpha ,\beta \right)\;=\;\sum_{i=1}^{N}\text{log}\;P\left(x_{i};\alpha ,\beta \right)\;=\;\left(\alpha -1\right)X\;+\;\left(\beta -1\right)Y\;-\;N\text{log}B\left(\alpha ,\beta \right), \end{array}$$

with $\left(X\;=\;\sum_{i=1}^{N}\text{log}\;x_{i},Y\;=\;\sum_{i=1}^{N}\text{log}\left(1-x_{i}\right)\right)$. In a Bayesian setting typically to avoid the dirac-like shape of the beta distribution (see discussion in Sect. 4.2), a prior law can be added:

$$\begin{array}{c} P\left(\alpha ,\beta \right)\;\propto \;B{\left(\alpha ,\beta \right)}^{K}e^{-a\alpha }e^{-b\beta }\;\text{for}\;K<N,a,b\in R^{+}. \end{array}$$

The objective function of the MAP estimator becomes:

$$\begin{array}{c} f^{*}\left(\alpha ,\beta \right)=\alpha \left(X\;\;-\;\;a\right)\;\;+\;\;\beta \left(Y\;\;-\;\;b\right)\;\;-\;\;\left(N\;\;-\;\;K\right)\text{log}\;B\left(\alpha ,\beta \right) \end{array}$$ (11)

with inequality constraints *g*_1_ = *ϵ* − *α* ≤ 0 and *g*_2_ = *ϵ* − *β* ≤ 0. *ϵ* > 0 is close to zero to form a closed space. Note that these constraints are affine functions and satisfy the regularity conditions. We thus search the solution of:

$$\begin{array}{c} F_{1}\left(z\right)=\left(X-a\right)-\left(N-K\right)(\psi \left(\tilde{\alpha }\right)-\psi \left(\tilde{\alpha }+\tilde{\beta }\right))+{\tilde{\mu }}_{1}=0, \end{array}$$

$$\begin{array}{c} F_{2}\left(z\right)=\left(Y-b\right)-\left(N-K\right)(\psi \left(\tilde{\beta }\right)-\psi \left(\tilde{\alpha }+\tilde{\beta }\right))+{\tilde{\mu }}_{2}=0, \end{array}$$

$$\begin{array}{c} \epsilon -\tilde{\alpha }\leq 0,\;{\tilde{\mu }}_{1}\geq 0,\;F_{3}\left(z\right)={\tilde{\mu }}_{1}\left(\epsilon -\tilde{\alpha }\right)=0, \end{array}$$ (12)

$$\begin{array}{c} \epsilon -\tilde{\beta }\leq 0,\;{\tilde{\mu }}_{2}\geq 0,\;F_{4}\left(z\right)={\tilde{\mu }}_{2}\left(\epsilon -\tilde{\beta }\right)=0, \end{array}$$ (13)

where *ψ*(⋅) represents the di-gamma function.

We apply the Newton Raphson method on *F*(***z***) = [*F*_1_, *F*_2_, *F*_3_, *F*_4_]^*t*^ with:

$$\begin{array}{c} z^{\left(n+1\right)}-z^{\left(n\right)}=-J{\left(z^{\left(n\right)}\right)}^{-1}F\left(z^{\left(n\right)}\right), \end{array}$$ (14)

and checking the inequality constraints in (12) and (13).

We next show that **J**(***z***) is always invertible given the inequality constraints. The $J\left(z\right)=\left[\frac{\partial F_{i}}{\partial z_{j}}\right]$ writes:

$$\begin{array}{c} J\left(z\right)=[\begin{array}{cc} \left(N-K\right)[\begin{array}{cc} \psi^{\left(1\right)}\left(\alpha +\beta \right)-\psi^{\left(1\right)}\left(\alpha \right) & \psi^{\left(1\right)}\left(\alpha +\beta \right)\\ \psi^{\left(1\right)}\left(\alpha +\beta \right) & \psi^{\left(1\right)}\left(\alpha +\beta \right)-\psi^{\left(1\right)}\left(\beta \right) \end{array}] & I_{2}\\ \begin{array}{cc} -\mu_{1} & 0\\ 0 & -\mu_{2} \end{array} & \begin{array}{cc} -\alpha & 0\\ 0 & -\beta \end{array} \end{array}] \end{array}$$

where *ψ*^(1)^(⋅) is the tri-gamma function (second derivative of the log-gamma). Its determinant is:

$$\begin{array}{c} \text{det}\;J(z)=\text{det}\;[\begin{array}{cc} A & B\\ C & D \end{array}]=\text{det}\;AD-BC={\left(N-K\right)}^{2}\alpha \beta \\ \left(\psi^{\left(1\right)}\left(\alpha \right)\psi^{\left(1\right)}\left(\beta \right)-\left(\psi^{\left(1\right)}\left(\alpha \right)+\psi^{\left(1\right)}\left(\beta \right)\right)\psi^{\left(1\right)}\left(\alpha +\beta \right)\right)\\ +\left(N-K\right)\mu_{1}\beta \left(\psi^{\left(1\right)}\left(\beta \right)-\psi^{\left(1\right)}\left(\alpha +\beta \right)\right)\\ +\left(N-K\right)\mu_{2}\alpha \left(\psi^{\left(1\right)}\left(\alpha \right)-\psi^{\left(1\right)}\left(\alpha +\beta \right)\right) \end{array}$$

The second equality is due to the fact that *C* and *D* commute (i.e. *CD* = *DC*). From the relation

$$\begin{array}{c} \psi^{\left(1\right)}\left(\alpha \right)\psi^{\left(1\right)}\left(\beta \right)>\left(\psi^{\left(1\right)}\left(\alpha \right)+\psi^{\left(1\right)}\left(\beta \right)\right)\psi^{\left(1\right)}\left(\alpha +\beta \right) \end{array}$$

it can be verified that det **J**(***z***) > 0. Thus the Jacobian matrix is always invertible.

## B Kulback-Leiber divergence for generalized normal distributions

The probability density of the generalized normal distribution writes:

$$\begin{array}{c} P\left(x;\alpha ,\beta ,\mu \right)=\frac{\beta }{2\alpha \Gamma (1/\beta )}\;e^{-{\left(|x-\mu |/\alpha \right)}^{\beta }}\;\text{for}\;\alpha ,\beta >0 \end{array}$$

Thus, the Kullback-Leibler divergence is:

$$\begin{array}{c} D_{\text{KL}}\left(P\|Q\right)=\int_{R}\frac{\beta_{p}}{2\alpha_{p}\Gamma \left(1/\beta_{p}\right)}\;e^{-(|x-\mu_{p}{\left|/\alpha_{p}\right)}^{\beta_{p}}}\times \text{log}\;(\frac{\frac{\beta_{p}}{2\alpha_{p}\Gamma \left(1/\beta_{p}\right)}\;e^{-(|x-\mu_{p}{\left|/\alpha_{p}\right)}^{\beta_{p}}}}{\frac{\beta_{q}}{2\alpha_{q}\Gamma \left(1/\beta_{q}\right)}\;e^{-(|x-\mu_{q}{\left|/\alpha_{q}\right)}^{\beta_{q}}}})\;\text{d}x\\ =\text{log}\;(\frac{\beta_{p}\alpha_{q}\Gamma \left(1/\beta_{q}\right)}{\beta_{q}\alpha_{p}\Gamma \left(1/\beta_{p}\right)})+\int_{R}\frac{\beta_{p}}{2\Gamma (1/\beta_{p})}e^{-{\left(\frac{|x-\mu_{p}|}{\alpha_{p}}\right)}^{\beta_{p}}}(-{\left(\frac{|x-\mu_{p}|}{\alpha_{p}}\right)}^{\beta_{p}}+{\left(\frac{|x-\mu_{q}|}{\alpha_{q}}\right)}^{\beta_{q}})\;\frac{\text{d}x}{\alpha_{p}}. \end{array}$$

Let $t=\frac{x-\mu_{p}}{\alpha_{p}}\Leftrightarrow \text{d}x=\alpha_{p}\text{d}t$. Since *α*_*p*_ > 0, we have:

$$\begin{array}{c} D_{\text{KL}}\left(P\|Q\right)=\text{log}\;(\frac{\beta_{p}\alpha_{q}\Gamma \left(1/\beta_{q}\right)}{\beta_{q}\alpha_{p}\Gamma \left(1/\beta_{p}\right)})-\int_{R}\beta_{p}/2\Gamma \left(1/\beta_{p}\right)\cdot e^{-{\left|t\right|}^{\beta_{p}}}\;{\left|t\right|}^{\beta_{p}}\text{d}t\\ +\underset{\left(*\right)}{\underset{︸}{\int_{R}\beta_{p}/2\Gamma \left(1/\beta_{p}\right)\cdot e^{-{\left|t\right|}^{\beta_{p}}}\;(|t\alpha_{p}+\mu_{p}-\mu_{q}{\left|/\alpha_{q}\right)}^{\beta_{q}}\text{d}t}} \end{array}$$

Since Γ(*z* + 1) = *z* Γ(*z*), the second term can be further simplified:

$$\begin{array}{c} \int_{0}^{+\infty }\;\frac{\beta_{p}}{\Gamma (1/\beta_{p})}\;e^{-t^{\beta_{p}}}\;t^{\beta_{p}}\text{d}t=\frac{\Gamma (1/\beta_{p}+1)}{\Gamma (1/\beta_{p})}=\frac{1}{\beta_{p}} \end{array}$$

In the following, we treat the term in (*). First define $\tilde{u}=\frac{|\mu_{p}-\mu_{q}|}{\alpha_{p}}$ to replace (*) with:

$$\begin{array}{c} {\left(\frac{\alpha_{p}}{\alpha_{q}}\right)}^{\beta_{q}}\;\;\;\frac{\beta_{p}}{2\Gamma (1/\beta_{p})}\int_{R}\;\;e^{-{\left|t\right|}^{\beta_{p}}}{|t+\tilde{u}|}^{\beta_{q}}\text{d}t=k_{1}\;\;\int_{R}e^{-{\left|t\right|}^{\beta_{p}}}{|t+\tilde{u}|}^{\beta_{q}}\text{d}t\\ \overset{t^{\prime }=t/\tilde{u}}{=}k_{1}\;\;\int_{R}\;e^{-{\left(\tilde{u}t^{\prime }\right)}^{\beta_{p}}}{|\tilde{u}\left(t^{\prime }+1\right)|}^{\beta_{q}}\tilde{u}\text{d}t^{\prime }=k_{1}{\tilde{u}}^{\beta_{q}+1}\;\int_{R}\;e^{-{\tilde{u}}^{\beta_{p}}{\left|t\right|}^{\beta_{p}}}{|t+1|}^{\beta_{q}}\text{d}t\\ =k_{2}\;\int_{R}\;e^{-{\xi |t|}^{\beta_{p}}}{|t+1|}^{\beta_{q}}\text{d}t \end{array}$$

with $k_{1}={\left(\frac{\alpha_{p}}{\alpha_{q}}\right)}^{\beta_{q}}\frac{\beta_{p}}{2\Gamma (1/\beta_{p})}$, $\xi ={\tilde{u}}^{\beta_{p}}$ and $k_{2}=k_{1}\;{\tilde{u}}^{\beta_{q}+1}>0$.

Since both |*t*| and |*t* + 1| exist in the expression, we further decompose (*) into:

$$\begin{array}{c} k_{2}\int_{0}^{1}e^{-\xi t^{\beta_{p}}}[{\left(t+1\right)}^{\beta_{q}}+{\left(1-t\right)}^{\beta_{q}}]\text{d}t+k_{2}\int_{1}^{+\infty }e^{-\xi t^{\beta_{p}}}[{\left(t+1\right)}^{\beta_{q}}+{\left(t-1\right)}^{\beta_{q}}]\text{d}t \end{array}$$

Notice that when *β*_*q*_ is even, the two functions inside the integrals are identical since ${\left(t-1\right)}^{\beta_{q}}={\left(1-t\right)}^{\beta_{q}}$. For instance, if *β*_*q*_ = 2:

$$\begin{array}{c} k_{2}\int_{0}^{1}\;\;e^{-\xi t^{\beta_{p}}}2[t^{2}+1]\;\text{d}t+k_{2}\int_{1}^{+\infty }e^{-\xi t^{\beta_{p}}}2[t^{2}+1]\;\text{d}t\\ =2k_{2}\int_{0}^{\infty }e^{-\xi t^{\beta_{p}}}[t^{2}+1]\;\text{d}t=\frac{2k_{2}}{\beta_{p}}(\frac{\Gamma (3/\beta_{p})}{\xi^{3/\beta_{p}}}+\frac{\Gamma (1/\beta_{p})}{\xi^{1/\beta_{p}}}), \end{array}$$

by exploiting the relation:

$$\begin{array}{c} \int_{0}^{+\infty }e^{-\xi t^{\beta_{p}}}t^{k}\text{d}t=\Gamma (\frac{k+1}{\beta_{p}})/(\beta_{p}\;\xi^{\frac{k+1}{\beta_{p}}}). \end{array}$$ (15)

This result can be generalized for all even-valued *β*_*q*_ including *β*_*q*_ = 0 though it is supposed to be strictly positive by definition. Let’s then investigate the case of *β*_*q*_ = 2*n* + 1 with n∈N. First, for *β*_*q*_ = 1,

$$\begin{array}{cc} (*)= & k_{2}\int_{0}^{1}\;e^{-\xi t^{\beta_{p}}}\left(2\right)\text{d}t+k_{2}\int_{1}^{+\infty }\;e^{-\xi t^{\beta_{p}}}\left(2t\right)\text{d}t=2k_{2}[\int_{0}^{1}\;e^{-\xi t^{\beta_{p}}}\text{d}t+\int_{1}^{+\infty }\;e^{-\xi t^{\beta_{p}}}t\text{d}t]\\ \overset{y=\xi t^{\beta_{p}}}{=} & \frac{2k_{2}}{\beta_{p}}[{\left(1/\xi \right)}^{\frac{1}{\beta_{p}}}\;\;\;\int_{0}^{\xi }\;\;\;e^{-y}y^{\frac{1}{\beta_{p}}-1}\text{d}y+{\left(1/\xi \right)}^{\frac{2}{\beta_{p}}}\;\;\;\int_{\xi }^{\infty }\;\;\;e^{-y}y^{\frac{2}{\beta_{p}}-1}\text{d}y]\\ = & \left(2k_{2}/\beta_{p}\right)[{\left(1/\xi \right)}^{\frac{1}{\beta_{p}}}\;\gamma \left(1/\beta_{p},\xi \right)+{\left(1/\xi \right)}^{\frac{2}{\beta_{p}}}\;\Gamma \left(2/\beta_{p},\xi \right)] \end{array}$$

in which

$$\begin{array}{c} \gamma \left(s,x\right)=\int_{0}^{x}\;t^{s-1}\;e^{-t}\;\text{d}t,\;\Gamma \left(s,x\right)=\int_{x}^{\infty }\;\;t^{s-1}\;e^{-t}\;\text{d}t \end{array}$$

are the lower and upper incomplete gamma functions respectively. Similarly, for *β*_*q*_ = 3, we develop the (1 + *t*)^3^, (1 − *t*)^3^ and (*t* − 1)^3^ terms:

$$\begin{array}{c} \left(*\right)=k_{2}(\int_{0}^{1}\;\;e^{-\xi t^{\beta_{p}}}\left(2+6t^{2}\right)\text{d}t+\int_{1}^{+\infty }\;\;\;e^{-\xi t^{\beta_{p}}}\left(6t+2t^{3}\right)\text{d}t), \end{array}$$

to coerce (*) into a sum of lower and upper incomplete gamma functions. As in (15), we use the following relations:

$$\begin{array}{cc} \int_{0}^{1}e^{-\xi t^{\beta_{p}}}t^{k}\text{d}t & =\gamma (\frac{k+1}{\beta_{p}},\xi )/(\beta_{p}\xi^{\frac{k+1}{\beta_{p}}}), \end{array}$$ (16)

$$\begin{array}{cc} \int_{1}^{\infty }e^{-\xi t^{\beta_{p}}}t^{k}\text{d}t & =\Gamma (\frac{k+1}{\beta_{p}},\xi )/(\beta_{p}\xi^{\frac{k+1}{\beta_{p}}}). \end{array}$$ (17)

To generalize, (*) is a sum of either weighted gamma functions using (15) if *β*_*q*_ is even or a sum of weighted upper and lower incomplete gamma functions using (16) and (17) if *β*_*q*_ is odd. *β*_*q*_-degree binomial coefficients are used to calculate the weights. Next we show that (*) is monotonously increasing:

$$\begin{array}{c} \frac{\partial (*)}{\partial \beta_{q}}=k_{2}\int_{R}e^{-{\xi |t|}^{\beta_{p}}}{\left|1+t\right|}^{\beta_{q}}\;\text{log}\;\left|1+t\right|\text{d}t>0 \end{array}$$

in which *k*_2_ and $e^{-{\xi |t|}^{\beta_{p}}}{\left|1+t\right|}^{\beta_{q}}$ are positive for t∈R. log|1 + *t*| is negative for t ∈ [−2, 0], and positive otherwise. A sufficient condition is:

$$\begin{array}{c} \int_{-2}^{2}e^{-\xi t^{\beta_{p}}}{\left|1+t\right|}^{\beta_{q}}\;\text{log}\;\left|1+t\right|\;\text{d}t>0 \end{array}$$ (18)

By splitting the integral into 2 parts and letting *y* = −*t* in the first part, the integral of the above inequality becomes

$$\begin{array}{l} \overset{2}{\underset{0}{\int }}e^{-\xi y^{\beta_{p}}}{\left|1-y\right|}^{\beta_{q}}\text{log}\left|1-y\right|\text{d}y+\overset{2}{\underset{0}{\int }}e^{-\xi y^{\beta_{p}}}{\left(1+y\right)}^{\beta_{q}}\text{log}\left(1+y\right)\text{d}y\\ =\overset{2}{\underset{0}{\int }}e^{-\xi y^{\beta_{p}}}\underset{G_{\beta_{q}}\left(y\right)}{\underset{︸}{\left({\left|1-y\right|}^{\beta_{q}}\;\text{log}\left|1-y\right|+{\left(1+y\right)}^{\beta_{q}}\;\text{log}\left(1+y\right)\right)}}\text{d}y \end{array}$$

Note that the function $G_{\beta_{q}}\left(y\right)$ is a continuous function of *y*, differentiable by piece and $G_{\beta_{q}}\left(y\right)\geq 0$ for all *y* ∈ [0, 2], *β*_*q*_ > 0, which validates the condition in (18).

## C List of acronyms, abbreviations and symbols

**Table:** 

*a*	Raw amplitude
*c*	Absolute correlation
*C*(*t*)	Candidate selected at instant t
*C_i_*(*t*)	Feature i of the candidate selected at instant t
*D*(*t*)	Final detection decision at instant t
$D_{\text{KL}}^{i}$	Kullback-Leibler divergence of feature i
${\bar{D}}_{\text{KL}}^{i}$	Modified Kullback-Leibler divergence of feature i
I	Set of complementary features
λ	Decision threshold
$P_{i}(C_{i}(t);\Theta_{i0},H_{0})$	Probability density function of feature i corresponding to hypothesis $H_{0}$ with Θ_*i*0_ parameter(s)
$P_{i}(H_{1}|C_{i}(t))$	Posterior probability of validating $H_{1}$ for feature *C_i_*(*t*)
*s*	Squared slope
Θ	Parameter(s) of the probability density function set for hypothesis $H_{0}$
DER	Detection error rate
ECG	Electrocardiogram
FIR	Finite Impulse Response
FN	False Negative
FP	False Positive
GND	Generalized Normal Distribution
KLD	Kullback-Leibler Divergence
MAP	Maximum A Posterior
MFPD	Multi-Feature Probabilistic Detector
MLE	Maximum Likelihood Estimator
pdf	Probability Density Function
+P	Positive predictivity
SAecg	Signal use to extract amplitude feature
SCD	Spatiotemporal Characteristics Detector
Se	Sensitivity
SFecg	Signal use to extract slope feature
SFPD	Single Featured Probabilistic Detector
SNR	Signal-to-Noise Ratio
TN	True Negative
TP	True Positive
UNSW	University of New South Wales detector
WBD	Wavelet-Based Detector
